# Stroke aftercare in Germany: findings from an online survey in the outpatient setting of a neurovascular network

**DOI:** 10.3389/fneur.2025.1507003

**Published:** 2025-03-12

**Authors:** John-Ih Lee, Robin Jansen, Jan F. Cornelius, Hubert Schelzig, Bernd Turowski, Rüdiger J. Seitz, Til Menge, Philipp Albrecht, Sebastian Jander, Jan Sobesky, Hans-Jürgen von Giesen, Marcel Dihné, Lars Wojtecki, Tristan Kölsche, Sajjad Muhammad, Tobias Ruck, Sven G. Meuth, Michael Gliem

**Affiliations:** ^1^Department of Neurology, Medical Faculty and University Hospital Düsseldorf, Heinrich-Heine-University Düsseldorf, Düsseldorf, Germany; ^2^Department of Neurosurgery, Medical Faculty and University Hospital Düsseldorf, Heinrich-Heine-University Düsseldorf, Düsseldorf, Germany; ^3^Department of Vascular and Endovascular Surgery, Medical Faculty and University Hospital Düsseldorf, Heinrich-Heine-University Düsseldorf, Düsseldorf, Germany; ^4^Department of Diagnostic and Interventional Radiology, Medical Faculty and University Hospital Düsseldorf, Heinrich-Heine-University Düsseldorf, Düsseldorf, Germany; ^5^Department of Neurology, LVR-Klinikum Düsseldorf, Heinrich-Heine-University Düsseldorf, Düsseldorf, Germany; ^6^Department of Neurology, Maria Hilf Clinic, Mönchengladbach, Germany; ^7^Department of Neurology, Marienhospital Düsseldorf, Düsseldorf, Germany; ^8^Department of Neurology, Johanna-Etienne-Hospital, Neuss, Germany; ^9^Department of Neurology, Maria Hilf Clinic, Krefeld, Germany; ^10^Department of Neurology, St. Lukas Klinik, Solingen, Germany; ^11^Departmemt of Neurology and Neurorehabilitation, Hospital Zum Heiligen Geist, Academic Teaching Hospital of the Heinrich-Heine-University Duesseldorf, Kempen, Germany; ^12^Institute of Clinical Neuroscience and Medical Psychology, Medical Faculty, Heinrich Heine University, Duesseldorf, Germany

**Keywords:** stroke, neurorehabiliation, aftercare and long term care, neurovascular, outpatient care

## Abstract

The evidence-based acute treatment of stroke patients in Germany is carried out according to standardized algorithms in more than 300 certified stroke units, and its quality is repeatedly assured by the German Stroke Society (DSG) and others. However, nationally structured and uniform stroke aftercare programs are missing, despite evidence that they contribute to the success of rehabilitation and improvement of everyday life. We used a 27-item online questionnaire, which was mailed to 4,195 outpatient physicians in the catchment area of the neurovascular network Neurovascular Network North Rhine plus (NEVANO+) located in the western part of Germany to assess actual structures of stroke aftercare, identify barriers, and possible solutions. Based on 152 completed anonymous answers to the questionnaire, a descriptive evaluation revealed that general practitioners and neurologists are seen to be responsible for stroke aftercare. Important improvement aspects, among others, were identified in intersectoral cooperation, the use of a post-stroke checklist, and connections to local self-help organizations. Stroke units could play a key role in stroke aftercare by providing these checklists, connecting self-help organizations, and offering education and coaching for supportive coordinating staff. Furthermore, existing neurovascular networks can be expanded to include rehabilitation clinics, geriatric clinics, and outpatient physicians to improve intersectoral communication, collaboration, and post-stroke care. Further studies should investigate whether intersectoral cooperation, checklists, and cooperation with self-help organizations within an extended neurovascular network have a positive impact on stroke aftercare and patients’ quality of life.

## Background

Stroke remains the second leading cause of death and the third leading cause of death and disability combined in the world (1). In Germany, first-ever stroke events affect approximately 196,000 people per year (2). Stroke accounts for approximately 63,000 deaths yearly, making it the third most frequent cause of death in Germany (2).

Although Germany’s evidence-based acute treatment of stroke patients in more than 300 certified stroke units follows standardized algorithms, with its quality repeatedly assured by the German Stroke Society (DSG) and others (3), there is a lack of nationwide, structured, and uniform stroke aftercare programs following rehabilitation. Furthermore, after discharge from the rehabilitation clinic, many stroke patients fall into a medical care gap. Contributing factors include the heterogeneity of the outpatient sector in Germany, insufficient involvement of neurological and psychiatric expertise, and the lack of reimbursement for a post-stroke disease management program. However, there is a clear need, as motor disabilities, cognitive disorders, depression, and occasionally epilepsy, anxiety, and fatigue are among the long list of relevant post-stroke sequelae that affect the daily lives of many patients (4, 5). Approximately 20% of them will suffer a second stroke in the 5 years following their first stroke, which is often avoidable through appropriate prevention (6–9).

In 2020, the DSG founded a commission on stroke aftercare. The aim was to evaluate the current situation of long-term aftercare and suggest improvements to its structure. Position papers regarding stroke aftercare were published (10–12). Clearly defined and consistent aftercare programs for patients after stroke are desirable as they contribute to the sustainability of achieving rehabilitation success (13) and improve quality of life (14).

Important objectives after a stroke are maintaining personal independence, avoiding the need for care, and, if necessary, facilitating professional reintegration into everyday working life. This requires, among other things, a seamless transition between high-quality inpatient and outpatient care, as good and continuous treatment following inpatient care has been shown to benefit patients (15). For optimal care, interprofessional cooperation is essential (16). However, this is often not part of the routine in outpatient care (17). Busse et al. (18) reported on acute stroke care in Germany with stroke units and the establishment of interdisciplinary neurovascular networks. These neurovascular networks aim to connect regional and transregional inpatient stroke units to bundle the expertise located there. Further integration of intersectoral cooperation between inpatient and outpatient care into these networks may provide further benefits. A pilot project in another part of Germany described the methodological challenges of evaluating a regional population-based integrated care system in Germany (19). Our neurovascular network, called Neurovascular Network North Rhine Plus (NEVANO+), was certified as a neurovascular network by the DSG in 2023. Located in western Germany, NEVANO+ includes stroke units certified as neurovascular networks, rehabilitation clinics, geriatric clinics, and outpatient neurologists. The network spans cities such as Düsseldorf, Mönchengladbach, Neuss, Solingen, Krefeld, and Kempen, along with parts of counties like Rhein Kreis Neuss, Heinsberg, Viersen, Kleve, Wesel, and Mettmann in North Rhine-Westphalia, covering almost 2.8 million inhabitants and providing stroke care for approximately 7,000 stroke and TIA patients per year.

Patients or their relatives usually need to take the initiative, often with the assistance of their general practitioner, to continue treatment independently (20). To continue the required therapies after the end of rehabilitation, patients have to find a therapist close to their home and organize appointments themselves. In other countries, for example, Sweden, a different approach has been established. Primary care centers exist, in which physicians, physiotherapists, occupational therapists, and psychologists work together in shared patient-centered interprofessional processes to improve the effectiveness of care (21). As the connection between acute and aftercare of stroke patients is crucial, the aim of this cross-sectional study was to gather the opinions of physicians responsible for the outpatient care of stroke patients about the current care and aftercare, to identify barriers, and to discuss possible solutions.

## Methods

The anonymous online survey comprising 27 questions was addressed to outpatient physicians outside the hospital and included general practitioners, specialists in internal medicine, neurologists, ophthalmologists, cardiologists, vascular surgeons, and other internal medicine subspecialists located in a part of western Germany belonging to the NEVANO+ area. The online survey was conducted in the period from 07 March 2023 to 07 April 2023. A total of 4,195 physicians were asked to participate by postal mail.

Survey validity: By creating a questionnaire for our own peer group of stroke care physicians, we could assume high face and content validity. The data described in the text were taken directly from the questionnaire and not transformed into a theoretical construct; therefore, construct validity was not applicable. The aims and target population were clearly identified. By choosing structured questions that were reevaluated and discussed by our local ethics committee and three experienced stroke physicians with expertise in outpatient management, we aimed to develop questions with strong validity. As the questions were informative and not predictive, predictive validity could not be assessed. Discussion of the findings within the neurovascular network of physicians caring for outpatient stroke patients re-confirmed the results.

Survey reliability: The survey was designed to have good reliability by addressing a topic in which the participants are stakeholders. The questionnaire was easy to use but required intentional online access. Participants answered questions about their daily business, and the majority of the questions (23 out of 27) were closed-ended.

Measures to increase the response rate: To increase the response rate, we designed a clear questionnaire with a simple layout, explained the aims of the study in an extra letter, made the questionnaire available online, and issued a reminder to complete the questionnaire.

A descriptive evaluation was carried out, which is presented in a combination of quantitative and qualitative data. For this purpose, relative and absolute frequencies for the items were calculated.

## Results

We received a total of 152 completed questionnaires, resulting in a response rate of 3.6%. A total of 88 male and 64 female physicians participated in this anonymous online survey. The majority of the physicians (60; 40%) were between 51 and 60 years old. General practitioners accounted for 62 (41%) of the survey participants, the most of any specialties. A total of 1770 general practitioners were contacted with a response rate of 3.8% (68 responses), 61 vascular surgeons with a response rate of 9.8% (6 responses), 1,248 internists with a response rate of 3.8% (48 total), 202 cardiologists with a response rate of 2.9% (6 responses), 272 neurologists with a response rate of 6.25% (17 responses), and 442 ophthalmologists with a response rate of 2.5% (11 responses).

**Figure 1:**
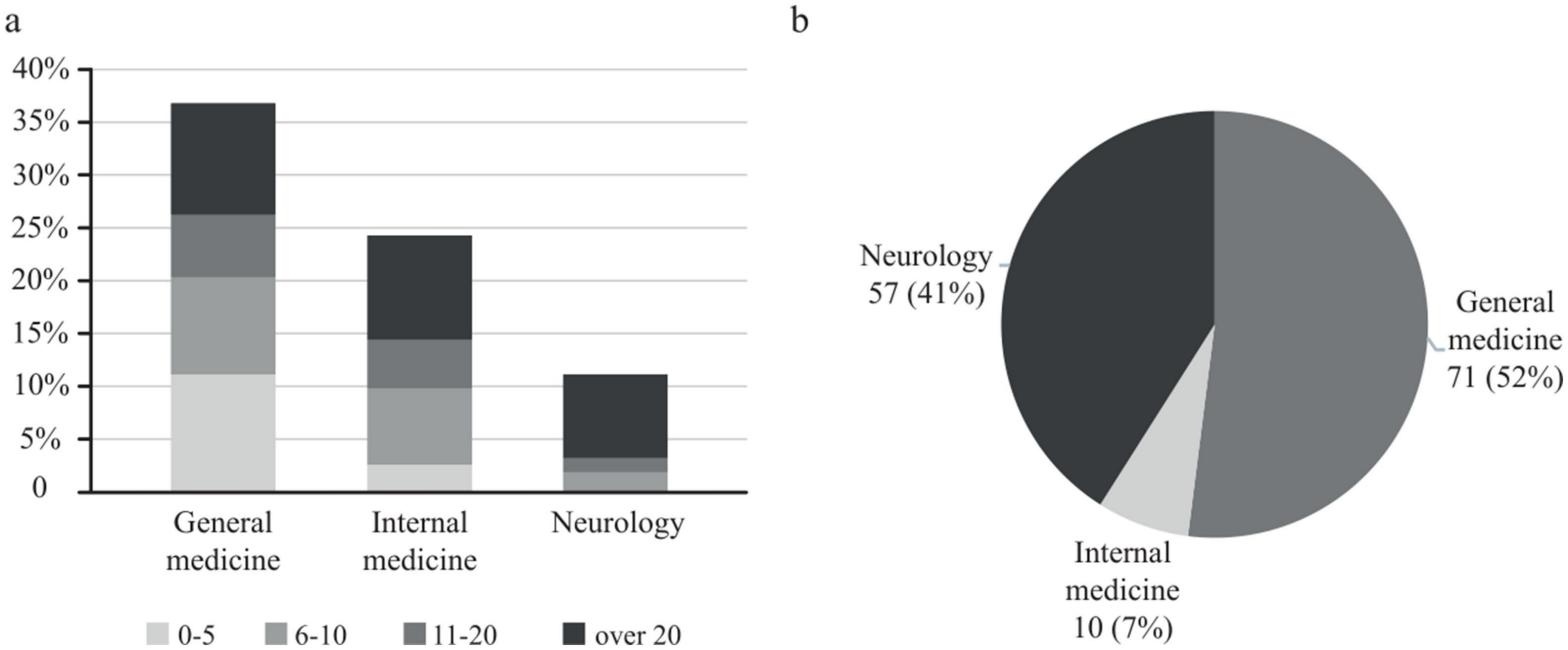
**(A)** Answers to the question: “How many after stroke-patients do you treat in a three-month period (0–5, 6–10, 11–20, or over 20 patients)?” The results are presented as percentages of all answers for the three most frequent specialties of outpatient physicians who responded to the survey. **(B)** Answers to the question: “Which specialty should mainly take care of stroke patients in the outpatient sector after they have left the hospital or rehabilitation?” Data are presented as absolute numbers and percentages of the outpatient physicians’ specializations.

General practitioners, internal medicine physicians, and neurologists were among the specializations with the highest number of stroke patients per physician (> 20 per 3 months) in aftercare (Figure 1A).

When asked which specialty should be in charge of stroke aftercare, general practitioners answered that general practitioners and neurologists should primarily treat stroke patients in aftercare (Figure 1B). Interestingly, internal medicine practitioners favored neurologists, general practitioners, and, to a smaller degree, themselves, whereas neurologists primarily saw themselves as responsible for stroke aftercare. In conclusion, the perception is that general practitioners and neurologists are in charge of stroke aftercare. The availability of office-based neurologists specialized in outpatient stroke care was rated by 27 (26%) participants as “very good” or “good,” by 38 (37%) as “neutral,” and by 39 (37%) as “very poor” or “poor.”

The majority of outpatient physicians receive their post-stroke patients from the stroke unit, inpatient neurology clinic, or rehabilitation clinic. The flow of information, at least between the stroke unit and the outpatient physicians, leaves room for improvement, with only 46 (35%) answers in the “very good” or “good” range and only 64 (52%) assessing the quality of the medical report as “very good” or “good.” In our survey, we asked whether the implementation of medical reports’ recommendations was possible. A total of 9 (7%) participants responded “strongly agree,” 48 (34%) responded “agree,” 64 (45%) responded “neutral,” and 20 (14%) responded “disagree” or “strongly disagree.” Regarding medical discharge letters, 25 (19%) of outpatient physicians answered that they always or frequently had queries about diagnosis, 33 (26%) about medication, and 49 (36%) about aftercare, such as about rehabilitation and medical supplies.

Potential areas of improvement in stroke aftercare, among others, included improved intersectoral cooperation (107; 34%) and the use/ offer of a post-stroke checklist (98;31%). The high interest in intersectoral cooperation was underlined by the fact that more than one-third (56; 39%) of the outpatient physicians were interested in joining a neurovascular network.

A total of 42 (32%) of the physicians assessed the availability of speech therapists specializing in aftercare stroke in the NEVANO+ area as “very good” or “good,” while 46 (35%) regarded this availability as “poor” or “very poor.”

Similarly, 47 (37%) physicians rated the availability of occupational therapy specializing in stroke aftercare as “very good” or “good” and as “poor” or “very poor.”

**Figure 2:**
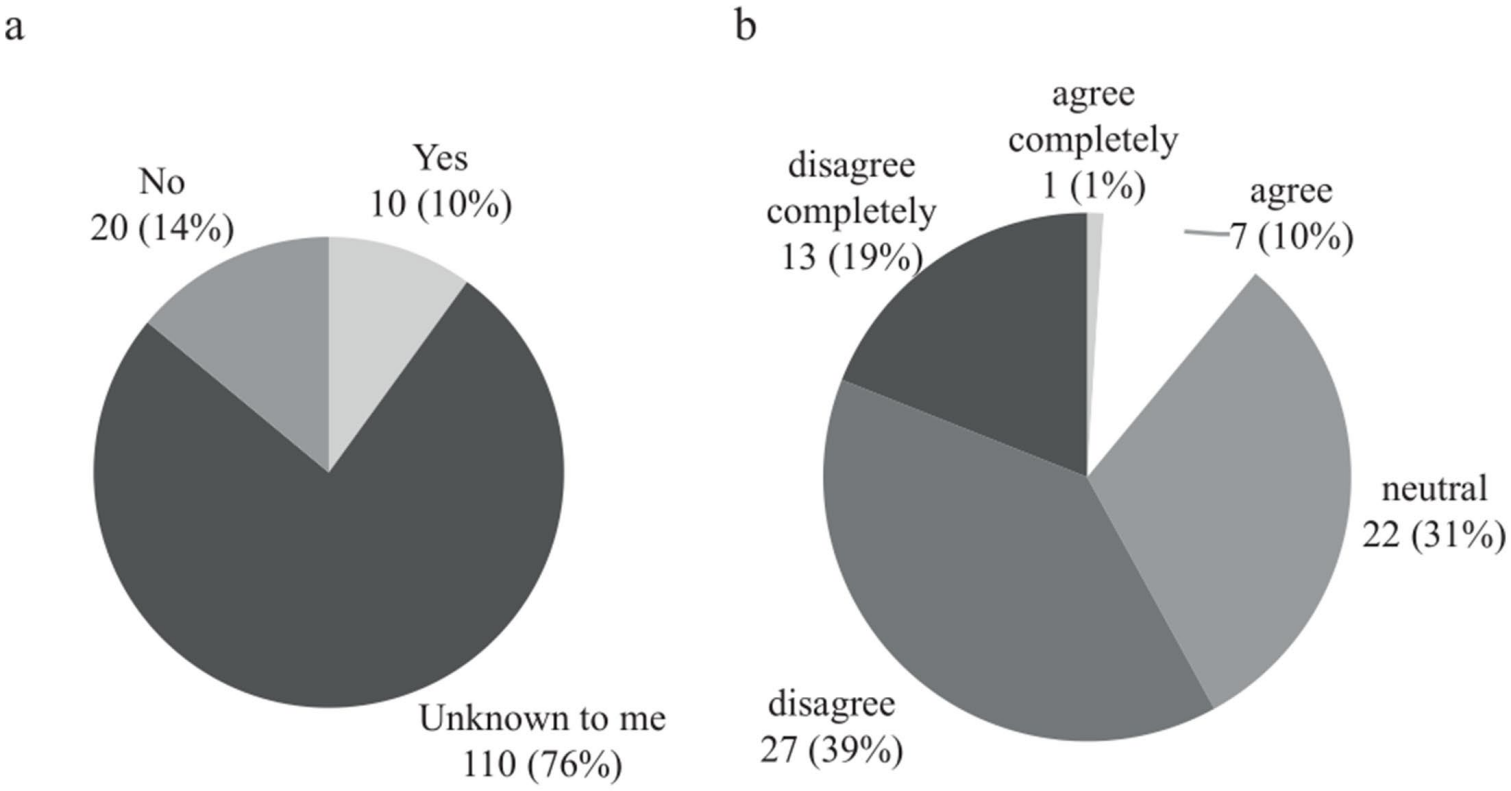
**(A)** Answers to the question of whether outpatient physicians are aware of local networks or structures that support stroke patients and their relatives. Data are presented as absolute numbers and percentages. **(B)** Opinion of outpatient physicians on whether they receive effective support in the management of post-stroke patients. Data are presented as absolute numbers and percentages.

The evaluation of physical therapy showed higher ratings, with 62 responses (47%) indicating “very good” or “good,” while only 33 responses (25%) indicated “poor” or “very poor.” Interestingly, only 18 (17%) of all outpatient physicians regarded the service of self-help organizations as “very good” or “good.” Only 14 respondents (10%) were aware of local services for stroke patient aftercare was known by Figure 2A, and 40 (58%) disagreed or completely disagreed with receiving effective support in the management of post-stroke patients (Figure 2B).

**Figure 3:**
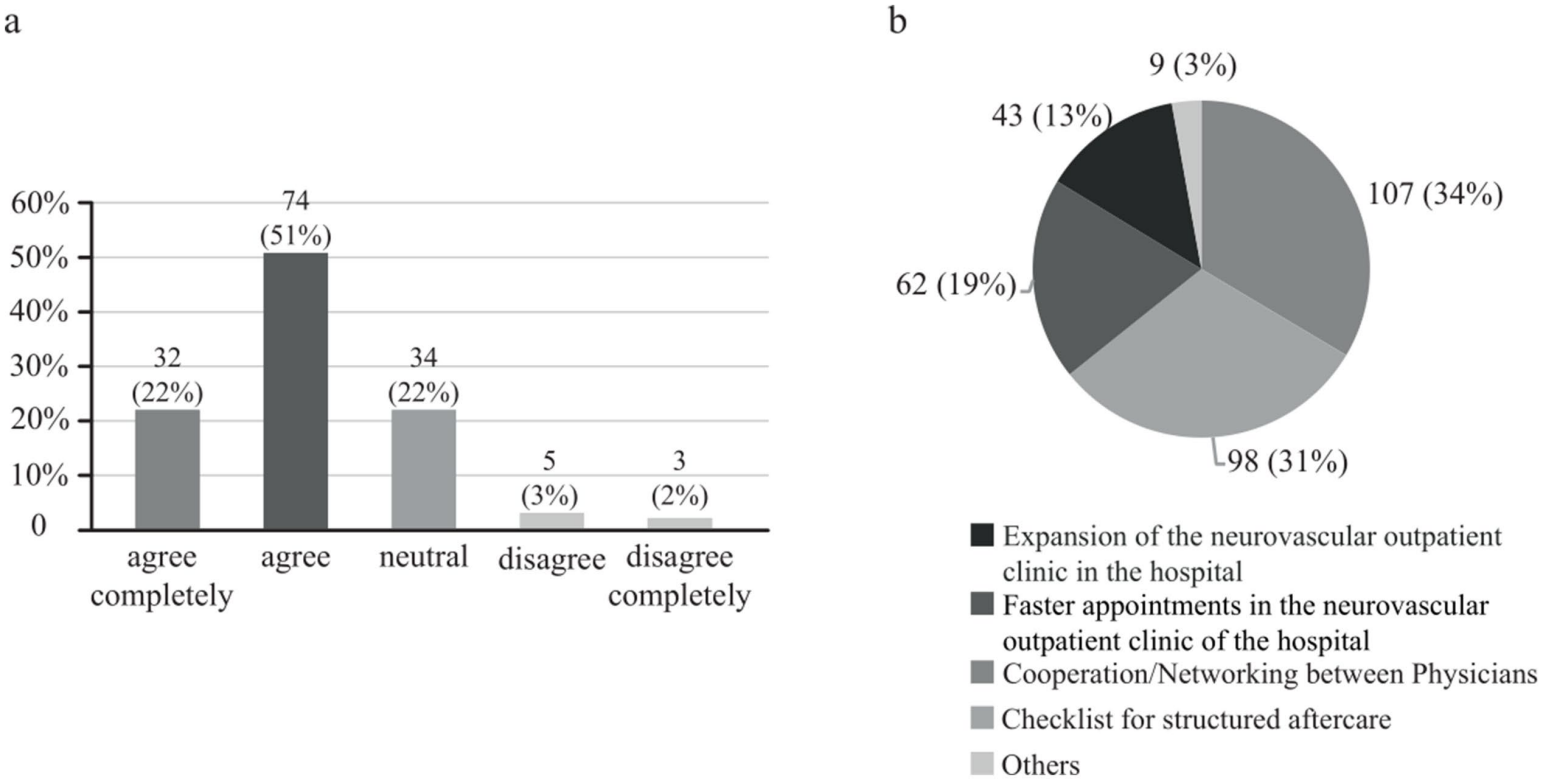
**(A)** Opinion of outpatient physicians on whether the stroke unit should be more involved as a competence center in stroke education. Data are presented as absolute numbers and percentages. **(B)** Opinion of outpatient physicians on what would be the best way to help stroke patients in aftercare. Multiple responses were possible. Data are presented as absolute numbers and percentages.

Regarding education, 106 physicians (72%) expressed a desire for increased involvement of the stroke unit in medical education (Figure 3A), while 92 (63%) favored a virtual format for medical education over traditional face-to-face learning courses. When considering the most effective approach to assist stroke patients in their post-stroke care, outpatient physicians considered cooperation/networking among physicians (107 (34%) answers), a checklist for structured aftercare (98 (31%)), faster appointments in the neurovascular outpatient clinic of a hospital (62 (19%)), and the expansion of the neurovascular outpatient clinic in a hospital as their main requests (Figure 3B). Multiple answers were allowed to this question.

## Discussion

The development of and evidence for the need for structured stroke aftercare is a hotly debated topic and requires further analysis. The positive outcome of the STROKE-CARD trial showed a reduction in cardiovascular disease and an improvement in quality of life in Austrian stroke or TIA patients and underlines the importance of structured stroke aftercare programs (22). On the other hand, the SANO trial showed no evidence of such improvements (23). In Germany, the results of the StroCare intervention study are currently awaited, which will expand existing stroke care with an extended approach that includes repeated outpatient visits to specialized stroke teams, the implementation of a case manager, the use of an electronic tool for communication between acute care, rehabilitation facilities, and outpatient care, and the definition of individualized treatment goals (24).

According to our survey, general practitioners and neurologists are seen to be mainly responsible for the follow-up of stroke patients. Thus far in Germany, the general practitioner is at the center of stroke follow-up care (4, 17), while a structured referral to a neurologist or stroke specialist in stroke aftercare is not established. This model allows a high degree of flexibility, but there is marked variability in access for patients to high-quality aftercare (10).

In addition, our survey confirmed the findings of previous studies such as Hempler et al.’s (4), who identified intersectoral communication and cooperation as a key issue for the successful transition of stroke patients from the inpatient to the outpatient sector. They discussed that treatment networks and standardized communication paths could help to improve intradisciplinary and interdisciplinary cooperation and communication in the aftercare system.

Therefore, an intra-sectoral and intersectoral network with structured communication and cooperation pathways might be helpful in stroke follow-up care.

Furthermore, the answers may imply a structure with a key role for stroke units also in stroke aftercare, although the time of the patient’s journey spent in a stroke unit is typically only a matter of days. The role of the stroke unit could be the coordination of stroke aftercare by providing appropriate checklists, connecting self-help organizations, offering post-stroke outpatient support from professional and voluntary aids [“Schlaganfall-Lotsen,” “Schlaganfall-Helfer”; (25)], and organizing continuing medical education. The integration of rehabilitation clinics, geriatric clinics, and outpatient physicians into existing neurovascular networks might be a first step toward structured intersectoral communication and cooperation pathways. Regarding this complex post-stroke care topic, the DSG has formulated position papers (10–12), proposing a flexible structure to ensure evidence-based treatment standards, related instruments for quality assurance, and a multidisciplinary and transsectoral regional care network.

A large prospective, open-label, cluster-randomized controlled trial (SANO trial) with a structured ambulatory post-stroke care program for outpatient aftercare failed to show differences between patients with ischemic stroke in the intervention group and control groups with regard to the incidence of vascular events after 1 year (23). However, it must be mentioned that a 1-year follow-up phase was probably insufficient to evaluate the efficacy of the program, and a prospective long-term follow-up study is being prepared by the authors. There are other pilot projects (10–12), trying to develop model solutions in stroke aftercare.

The upcoming evaluation of these projects will facilitate an evidence-based and detailed discussion about the possibilities of stroke aftercare. This will also include new transsectoral forms of care, such as the expansion of already existing stroke units or neurovascular networks into the outpatient sector. However, an adequate financing model with appropriate incentives would have to overcome the separate inpatient and outpatient sectors.

The limitations of our survey include the limited number of physicians who responded to our questionnaire, differences in stroke care experience due to years of experience, differences in specialty, non-response, sampling error, or framing bias. With regard to the latter, for example, it can be assumed that the question we asked about the need for intersectoral cooperation is more likely to be answered “yes,” as there are no disadvantages or additional workloads associated with improved cooperation. Another limitation is that all participants in the survey were physicians in the region of the NEVANO+ network. However, the gaps between stroke hospital care and outpatient management are common and often similar in many regions of the world.

## Conclusion

Existing neurovascular networks can be expanded to include rehabilitation clinics, geriatric clinics, and outpatient physicians to improve intersectoral communication, collaboration, and post-stroke care. Further studies should be conducted to investigate whether intersectoral cooperation within an extended neurovascular network has a positive impact on stroke aftercare and patient quality of life. The coordination of stroke aftercare across sectors may play a key role, and supportive staff in addition to physicians may be able to improve the care.

## Data Availability

The raw data supporting the conclusions of this article will be made available by the authors, without undue reservation.

## Group members of the NEVANO+

Medical Faculty and University Hospital Düsseldorf, Heinrich-Heine-University Düsseldorf, Germany (Coordinating Center): Department of Neurology, Sven G. Meuth (Head), Tobias Ruck, Michael Gliem, John-Ih Lee. Department of Neurosurgery, Jan F. Cornelius, Sajjad Muhammad. Department of Vascular and Endovascular Surgery, Hubert Schelzig. Department of Diagnostic and Interventional Radiology, Bernd Turowski. LVR-Klinikum Düsseldorf: Department of Neurology, Til Menge (Head). Maria Hilf Clinic Mönchengladbach: Department of Neurology, Philipp Albrecht (Head). Department of Vascular and Endovascular Surgery, Barbara Weis-Müller (Head). Department of Radiology and Neuroradiology, Adrian Ringelstein (Head). Marien Hospital Düsseldorf: Department of Neurology, Sebastian Jander (Head). Department of Diagnostic and Interventional Radiology, Stefan Diederich (Head). Maria Hilf Clinic Krefeld: Department of Neurology, Hans-Jürgen von Giesen (Head). Hospital zum Heiligen Geist Kempen: Department of Neurology, Lars Wojtecki (Head). Johanna-Etienne-Krankenhaus Neuss: Department of Neurology, Jan Sobeskäy (Head). St. Lukas Klinik Solingen: Department of Neurology, Marcel Dihné (Head). Department of Neuroradiology (radprax), Hannes Nordmeyer (Head). St. Mauritius Therapieklinik Meerbusch: Department of Neurology, Mathias Göppert (Head). Mediclin Rhein-Ruhr Essen Kettwig: Department of Neurology, Saskia Meves (Head). Vamed Klinik Hagen Ambrock: Specialist clinic for neurological and neurosurgical rehabilitation, Christoph Schäfer (Head). Vamed Klinik Hattingen: Rehabilitation center for Neurology, Neurosurgery, Neuropediatrics, Axel Petershofer (Head). Lukas Klinikum Neuss: Department of Geriatrics, Oliver Franz (Head). St. Martinus Krankenhaus: Department of Geriatrics, Albert Lukas (Head). Kreiskrankenhaus Grevenbroich St. Elisabeth: Department of Geriatrics, Oliver Franz (Head). Elisabeth Krankenhaus, Städtische Kliniken Mönchengladbach: Department of Geriatrics, Thomas Jaeger (Head). Krankenhaus Maria-Hilf Krefeld: Department of Geriatrics, Frau Jasna Katsaounis-Juras (Head). Neuropraxis Lindemann3 Düsseldorf, Derksen. Neuro-Centrum Düsseldorf, Ralph Ewerhart, Stefan Jun Groiss, Hans-Werner Nelles, Beutler, Kirsten Erdmann, Elke Schröder, and Gabriele Arendt.
